# Enhanced stimulation of human tumor-specific T cells by dendritic cells matured in the presence of interferon-γ and multiple toll-like receptor agonists

**DOI:** 10.1007/s00262-017-2029-4

**Published:** 2017-06-10

**Authors:** Tanja Lövgren, Dhifaf Sarhan, Iva Truxová, Bhavesh Choudhary, Roeltje Maas, Jeroen Melief, Maria Nyström, Ulrika Edbäck, Renee Vermeij, Gina Scurti, Michael Nishimura, Giuseppe Masucci, Alex Karlsson-Parra, Andreas Lundqvist, Lars Adamson, Rolf Kiessling

**Affiliations:** 1 Department of Oncology-Pathology, Cancer Center Karolinska, Karolinska Institutet, Stockholm, Sweden; 2 Masonic Cancer Center, University of Minnesota, Minneapolis, MN USA; 3 Department of Surgery, Loyola University Chicago, Maywood, IL USA; 4 Department of Immunology, Genetics and Pathology, Uppsala University, Uppsala, Sweden; 5 Cancer Center Karolinska R8:01, Karolinska Universitetssjukhuset Solna, 171 76 Stockholm, Sweden

**Keywords:** Cancer, Dendritic cell-vaccine, IFNγ, R848, Poly I:C, LPS

## Abstract

Dendritic cell (DC) vaccines have been demonstrated to elicit immunological responses in numerous cancer immunotherapy trials. However, long-lasting clinical effects are infrequent. We therefore sought to establish a protocol to generate DC with greater immunostimulatory capacity. Immature DC were generated from healthy donor monocytes by culturing in the presence of IL-4 and GM-CSF and were further differentiated into mature DC by the addition of cocktails containing different cytokines and toll-like receptor (TLR) agonists. Overall, addition of IFNγ and the TLR7/8 agonist R848 during maturation was essential for the production of high levels of IL-12p70 which was further augmented by adding the TLR3 agonist poly I:C. In addition, the DC matured with IFNγ, R848, and poly I:C also induced upregulation of several other pro-inflammatory and Th1-skewing cytokines/chemokines, co-stimulatory receptors, and the chemokine receptor CCR7. For most cytokines and chemokines the production was even further potentiated by addition of the TLR4 agonist LPS. Concurrently, upregulation of the anti-inflammatory cytokine IL-10 was modest. Most importantly, DC matured with IFNγ, R848, and poly I:C had the ability to activate IFNγ production in allogeneic T cells and this was further enhanced by adding LPS to the cocktail. Furthermore, epitope-specific stimulation of TCR-transduced T cells by peptide- or whole tumor lysate-loaded DC was efficiently stimulated only by DC matured in the full maturation cocktail containing IFNγ and the three TLR ligands R848, poly I:C, and LPS. We suggest that this cocktail is used for future clinical trials of anti-cancer DC vaccines.

**Electronic supplementary material:**

The online version of this article (doi:10.1007/s00262-017-2029-4) contains supplementary material, which is available to authorized users.

## Introduction

Efforts to vaccinate cancer patients with preparations of naturally occurring DC from blood has shown promising clinical results [1, 2]. However, these primary DC are rare and therefore the majority of DC trials have been based on DC derived from monocytes ex vivo (Mo-DC). Monocytes are abundant in blood and large quantities of Mo-DC can be easily generated in culture for most individuals.

The FDA-approved DC-based vaccine (Sipuleucel-T, Provenge, Dendreon) consists of a leukapheresis product that has been enriched for DC precursors and loaded with a recombinant fusion protein of prostatic acid phosphatase (PAP) and GM-CSF. This cell product was shown to prolong the overall survival in asymptomatic or minimally symptomatic, metastatic, hormone-refractory prostate cancer patients by 4.1 months [3]. Unfortunately, the numerous other trials on Mo-DC vaccination conducted in different malignancies have had limited clinical success even though immunological responses were commonly reported in response to both unmutated tumor-associated antigens and mutated neoepitopes [4–7]. Thus, there is a need for more efficient Mo-DC vaccines and/or to combine Mo-DC vaccines with other therapies in cancer patients.

The in vitro generation of iDC from monocytes is most commonly stimulated by culturing in the presence of IL-4 and GM-CSF, but for triggering subsequent differentiation into mDC there are numerous different protocols. For an efficient DC-based cancer vaccine, generated mDC have to be able to home to lymphoid tissue and, once there, efficiently stimulate tumor-specific Th1-type CD4+ T cells and CD8+ CTL that are able to eliminate tumor cells. To achieve this, mDC should express the lymph node homing receptor CCR7, high levels of co-stimulatory molecules such as CD80, CD83, and CD86 and produce high levels of the Th1-skewing and CTL-stimulatory cytokine IL-12 [8–10].

For many years the gold standard for a maturation cocktail of DC vaccines contained TNFα, IL-1β, IL-6, and prostaglandin E2 (PGE2) [11]. However, these DC were shown to produce very little IL-12p70, which was attributed to the presence of PGE2 in the cocktail [12]. Thus, alternate cocktails without PGE2 were suggested. Most of these involve the addition of molecules containing pathogen- and/or damage-associated molecular patterns (PAMP and DAMP, respectively), which bind to pattern recognition receptors (PRR), such as TLR expressed by the DC. The TLR expression differs depending on the DC subtype and maturation stage. Monocyte-derived iDC have been reported to express several TLR, including TLR3, 4, and 8, receptors for double-stranded RNA (dsRNA), LPS, and single-stranded RNA (ssRNA), respectively [13–17]. In line with this, maturation cocktails containing a mixture of TNFα, IL1β, IFNα, IFNγ, and the TLR3 agonist poly I:C [9, 18], IFNγ and poly I:C [19], IFNγ and the TLR4 agonists LPS or its derivative monophosphoryl lipid A (MPLA) [19–23] or IFNγ and the TLR7/8 agonist R848 [19, 21] generated mDC with improved IL-12p70 production while still expressing CCR7. Notably, the addition of IFNγ [9, 19, 24, 25] or combinations of more than one TLR agonist [24, 26, 27], (poly I:C and LPS, poly I:C and R848, LPS and R848) or both [28] (IFNγ, poly I:C, and R848 in combination with CD40L) potentiated the IL-12p70 production. Furthermore, the addition of multiple TLR agonists also increased CCR7 expression [24, 26]. Therefore, we decided to study the T cell-activating capacity of DC matured with TNFα, IFNγ, and ligands for all three TLR expressed by Mo-DC (TLR3, 4, and 8), alone or in combination. The aim was to develop an improved DC vaccine by optimizing the maturation protocol to induce high IL-12p70 production, maintained CCR7 expression, and potent ability to activate tumor-specific T cells.

## Materials and methods

### Cell preparation and culture

PBMC were prepared from healthy blood donor buffy coats by Ficoll-Hypaque (GE Healthcare) density-gradient centrifugation. Purification of monocytes and T cells was performed by MACS (Miltenyi Biotec) using positive selection with CD14 microbeads or negative selection with pan T cell isolation kit, respectively, according to the manufacturer’s instructions. Retroviral transduction of T cells with a TCR specific for tyrosinase_368–376_ 370D peptide was performed as previously described [29, 30].

The HLA-A2-positive A375 cell lines were cultured in DMEM (LifeTechnologies) supplemented with FCS (10%), penicillin (100 U/ml; LifeTechnologies), and streptomycin (100 μg/ml; LifeTechnologies). Wild-type, tyrosinase-negative A375 cells and A375 cells transduced with SAMEN retrovirus encoding the Tyrosinase_368–376_ 370D epitope were used. The A375 cells were harvested and diluted to 1.5 × 10^8^/ml in GMP-grade CellGro^®^ serum-free DC medium (CellGenix) + human serum albumin (HSA; 1%; Octapharma). The cells were lysed by 6 cycles of freeze-thawing in liquid nitrogen and 56 °C water bath. The resulting lysate was passed through a 70 µM cell strainer (Falcon). Protein concentration was measured using NanoDrop™ (Thermo Fischer Scientific).

Monocytes were cultured in 10 cm petri dishes (Corning) at 2 × 10^6^ cells/ml in 10 ml CellGro^®^ serum-free DC medium and differentiation was triggered by the addition of IL-4 (20 ng/ml; PeproTech) and GM-CSF (100 ng/ml; PeproTech) for 48 h. The resulting immature DC were harvested and washed and thereafter cultured in 12-well plates (TPP^®^) at 5 × 10^5^ cells/ml, 2 ml/well in CellGro^®^ serum-free DC medium supplemented with IL-4 and GM-CSF as before and in addition combinations, as specified, of TNFα (20 ng/ml; PeproTech), IFNγ (1000 IU/ml; Imukin^®^, Boehringer Ingelheim), R848 (2.5 µg/ml; VacciGrade™, Invivogen), Hiltonol© (20 µg/ml; OncoVir), poly I:C (20 µg/ml; Sigma-Aldrich or GE Healthcare), high and low molecular weight poly I:C (20 µg/ml; InVivoGen), LPS (10 ng/ml; Sigma-Aldrich or VacciGrade™, InvivoGen), MPLA-SM (10 ng/ml; VacciGrade™, InVivogen), and A375 cell lysates (15 µg/ml) for 18 h to generate mature dendritic cells. Where specified, peptide loading was performed after maturation, on harvested and washed DC, with either 10 µg/ml tyrosinase_368–376_ 370D peptide (YMDGTMSQV; Peptide 2.0) or HCV NS3_1406–1415_ (KLVALGINAV; Peptide 2.0), as control peptide, in PBS for 1 h at 37 °C before thorough washing. In addition, the previously published “gold standard” [10 ng/ml TNFα, 10 ng/ml IL1β (CellGenix), 1000 IU/ml IL-6 (CellGenix), and 1 μg/ml PGE2 (Sigma-Aldrich)] [11] and “alpha-type 1 DC” [50 ng/ml TNFα, 25 ng/ml IL-1β, 3000 IU/ml IFNα (PBL assay science), 1000 IU/ml IFNγ, and 250 ng/ml Hiltonol©)] [9] maturation cocktails were used for comparison during the iDC to mDC differentiation step where indicated.

T cell co-cultures were performed in round-bottom 96-well plates (TPP^®^) using CellGro^®^ serum-free DC medium containing human AB serum (2%) and IL-2 (20 IU/ml; Proleukin, Novartis). The DC were harvested and washed prior to co-culture. A ratio of 1 DC (25,000 cells) to 4 T cells (100,000 cells) in 100 µl/well was used and the co-cultures were incubated for 4 days. The selected setup was chosen to achieve the highest possible IFNγ production without the need for medium exchange or splitting of wells. CD3/CD28 beads (LifeTechnologies) were used according to the manufacturer’s instructions.

### Immunoassays

ELISA for IFNγ and IL-12p70 (MabTech) were performed according to the manufacturer’s instructions. Standard curves were plotted as four-parameter sigmoidal curves and unknowns as well as p-values for linear regressions were calculated and plotted using GraphPad Prism (GraphPad).

Multiplex cytokine detection of 13 cytokines (IL1β, IL-6, IL-8, IL-10, IL-12p70, IFNα2, IFNβ, IFNλ1, IFNλ2/3, IFNγ, TNFα, IP10, GM-CSF) was performed using the LEGENDPlex™ Human Anti-Virus Response panel (Biolegend) according to the manufacturer’s instructions. The data were acquired on a NovoCyte (ACEA Biosciences) flowcytometer and analyzed using the provided LEGENDPlex 7.0 software. Heat maps were generated in Microsoft Excel (Microsoft).

### Flow cytometry

Surface staining of DC for CD14 (clone M5E2, BioLegend), CD80 (clone 2D10, BioLegend), CD83 (clone HB15e, BioLegend), CD86 (clone IT2.2, BioLegend), HLA-DR (clone L243, BioLegend), CCR7 (clone 150503, BD Biosciences), IL-15Rα (clone JM7A4, BioLegend), IL-15 (clone 34559, RD Systems), PD-L1 (clone MIH1, BD Biosciences), CD40 (clone 5C3, BD Biosciences), CD206 (clone 15-2, Biolegend), and DC-SIGN (clone 9E9A8, Biolegend) was performed for 20 min in the dark in PBS with HSA (1%) at 4 °C. Antibody concentration had been titrated for optimal signal-to-noise ratio. Data were acquired on a NovoCyte (ACEA Biosciences) or a BD™ LSR II (BD Biosciences) and analyzed using FlowJo Software (TreeStar) as geometric MFI or percent positive cells.

## Results

### Enhanced IL-12p70 production by dendritic cells matured in presence of IFNγ, R848, and poly I:C

**Fig. 1:**
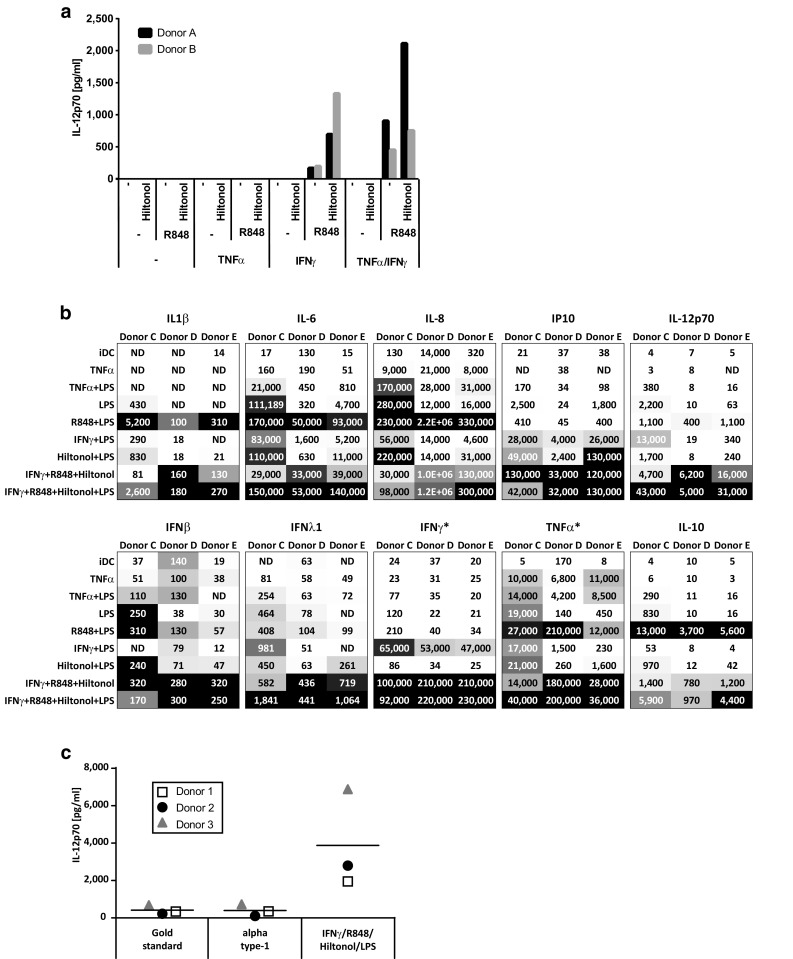
Production of cytokines by monocyte-derived dendritic cells matured in the presence of different stimulatory cocktails. Monocytes from two donors were screened for the production of IL-12p70 after an initial 48 h culture with GM-CSF/IL-4 followed by harvest, wash, and thereafter 18 h with GM-CSF/IL-4 together with different combinations of TNFα, IFNγ, R848, and the GMP-grade poly I:C Hiltonol^®^ (**a**). Monocytes from three donors were matured into mDC with the same stimulators as above and also LPS. The supernatants were screened for the production of a panel of different cytokines (**b**). Numbers represent concentrations (pg/ml) of the respective cytokine and the *gray scale* represents the lowest (*white*) to highest (*black*) concentrations within each donor. *Stars* indicate that the measured cytokine was added to some cocktails (~35,000 pg/ml IFNγ or 20,000 pg/ml TNFα). Monocytes from three donors were screened for the production of IL-12p70 after an initial 48 h culture with GM-CSF/IL-4 followed by 18 h with IFNγ, R848, Hiltonol, and LPS or with the “gold standard” (TNFα, IL1β, IL-6, PGE2) and “alpha type-1” (TNFα, IL-1β, IFNα, IFNγ, Hiltonol) DC cocktail (**c**)

In this study, we examined the effect of TNFα, IFNγ, and several TLR ligands independently or in combination on DC maturation. In a preliminary screening experiment, monocytes were purified from two healthy blood donors and used for maturation of dendritic cells. Throughout this study, the maturation was performed by a fast Mo-DC differentiation protocol, reported to result in DC with equal or better immune stimulatory capacity than previous longer protocols [23, 31–38]. Furthermore, we did a comparison with our previous 4 plus 2-day protocol and found no difference in DC viability or functionality (results not shown). Thus, monocytes were cultured for an initial 48 h in serum-free DC medium supplemented with GM-CSF and IL-4 followed by harvest and washing and thereafter 18 h in the same medium but with additional combinations of TNFα, IFNγ, the TLR7/8 agonist R848, and/or the GMP-grade poly I:C Hiltonol© before measuring IL-12p70 in the cultures (Fig. 1a). Notably, the maturation protocol with TNFα alone induced very little IL-12p70 production. The maturation cocktail containing IFN and R848 showed efficient IL-12p70 production, which was further increased after the addition of Hiltonol©. Adding TNFα to the other reagents had variable effect between the donors.

In an effort to further enhance the DC product, we evaluated the effect of additional TLR triggering by including the TLR4 agonist LPS in the DC maturation cocktails. Released cytokines were evaluated using a multiplex assay measuring 13 different cytokines of mainly Th1 type. Many of these (IL1β, IL-6, IL-8, IL-10, IL-12p70, IFNβ, IFNλ1, IP10, IFNγ, TNFα) were upregulated already in the IFNγ, R848, and Hiltonol©-matured DC compared to TNFα-matured DC (Fig. 1b). However, the production of most cytokines was further increased upon addition of LPS. Some were only weakly detected in any condition (IFNα2, IFNλ2/3; results not shown) and others have to be evaluated with caution since they were exogenously added to some conditions (IFNγ, TNFα, GM-CSF; results not shown for GM-CSF). However, higher TNFα levels were detected in supernatant from DC matured with IFNγ, R848, and Hiltonol with or without LPS, than in conditions where TNFα was added. Similarly, IFNγ was detected in greater levels in IFNγ, R848, and Hiltonol©-matured DC than the added amount.

Other cocktails generally induced much weaker cytokine induction than IFNγ, R848, and Hiltonol©-containing ones (with or without LPS). An exception was the combination of R848 and LPS. However, the R848 and LPS mixture induced much lower levels of IL-12p70 and IP10 while it induced the highest level of anti-inflammatory IL-10. The IL-10 level was upregulated also in IFNγ, R848, and Hiltonol©-matured DC (with or without LPS) but only modestly compared to pro-inflammatory cytokines/chemokines, such as IL6, IL-8, IP-10, and IL-12p70. Thus, for activating anti-tumor T cell responses, the combination of IFNγ, R848, Hiltonol©, and LPS displayed the most promising cytokine profile. To confirm the findings, the IFNγ, R848, Hiltonol©, and LPS combination was further compared to two previously published and commonly used DC maturation cocktails; the “gold standard DC” (TNFα, IL1β, IL-6, PGE2) [11] and the “alpha type-1 DC” (TNFα, IL-1β, IFNα, IFNγ, Hiltonol) [9] cocktails. None of these were induced as high levels of IL12p70 as DC cultures matured with IFNγ, R848, Hiltonol©, and LPS (Fig. 1c).

### GMP-grade poly I:C Hiltonol is as effective as non-GMP-grade poly I:C to induce IL-12p70 production in DC

**Fig. 2:**
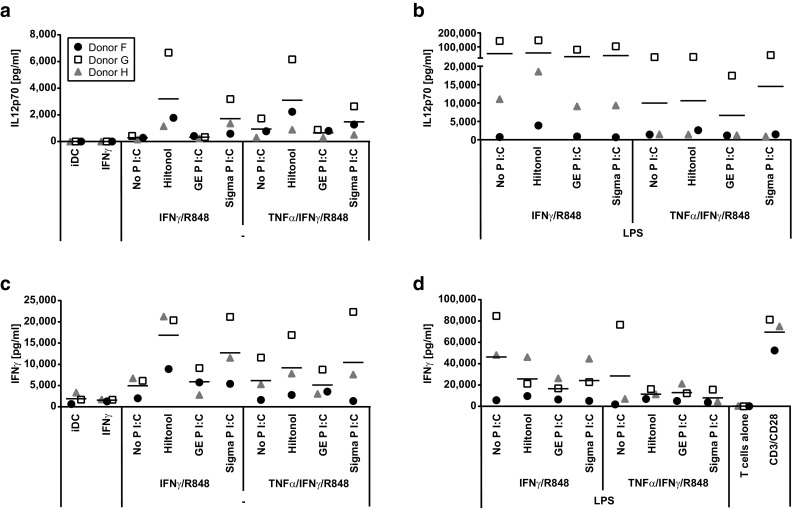
Activation of allogeneic T cells by monocyte-derived dendritic cells matured in the presence of different stimulatory cocktails. Monocytes from three donors were analyzed for the production of IL-12p70 after an initial 48 h culture with GM-CSF/IL-4 followed by harvest, wash, and thereafter 18 h with GM-CSF/IL-4/IFNγ/R848 together with combinations of TNFα and GMP-grade poly I:C Hiltonol^®^ or non-GMP-grade poly I:C from either GE Healthcare or Sigma-Aldrich without (**a**) or with addition of LPS (**b**). The monocyte-derived dendritic cells were harvested, washed, and then co-cultured for 4 days with allogeneic bulk T cells before the analysis of IFNγ production (**c**, **d**). Note the difference in scales of *y*-axes

To further assess the effect of poly I:C on DC IL-12p70 production, we studied the effect of different variants of poly I:C on DC maturation. Poly I:C is a heterogeneous product consisting of dsRNA of different length and the exact composition may differ between batches. Furthermore, structural changes may be introduced, for example to stabilize the product. Finally, non-GMP-grade poly I:C was occasionally found to be contaminated with LPS (results not shown). Hiltonol© is a GMP-grade, long-strand poly I:C, stabilized by a poly-lysine chain. Hiltonol was compared to two non-GMP-grade, mixed-length, unstabilized poly I:C for its ability to stimulate iDC when combined with IFNγ and R848. Hiltonol© and non-GMP-grade poly I:C from Sigma-Aldrich induced similar amounts of IL-12p70 while non-GMP-grade, unstabilized poly I:C from GE Healthcare induced much lower levels of IL-12p70 (Fig. 2a).

Further experiments showed no difference in IL-12p70 induction in DC when comparing low or high molecular weight poly I:C (results not shown). Addition of LPS to the maturation cocktail, on the other hand, strongly increased the IL-12p70 production (Fig. 2b). In contrast, addition of TNFα to maturation cocktails that lacked LPS had no effect (Fig. 2a) and decreased the IL-12p70-inducing capacity of maturation cocktails containing LPS (Fig. 2b). In summary, we conclude that the combination of IFNγ, R848, poly I:C, and LPS was the most efficient inducer of IL-12p70 production in DC and that GMP-grade poly I:C Hiltonol was as good or better than non-GMP-grade poly I:C.

### The ability of dendritic cells to activate allogeneic T cells correlates with their production of IL-12p70

**Fig. 3:**
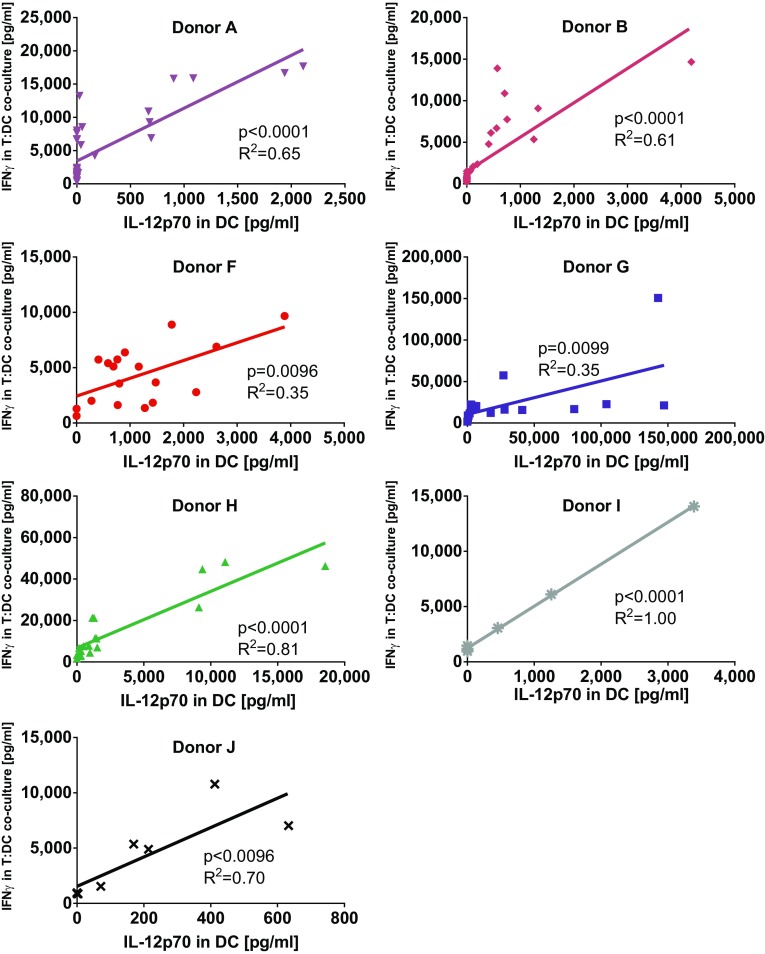
Correlation between IL-12p70 production in dendritic cells and IFNγ production by allogeneic bulk T cells stimulated with these dendritic cells. The amount of IL-12p70 produced by monocyte-derived dendritic cells matured by different maturation cocktails was compared to their ability to, after harvest and wash, induce IFNγ production in allogeneic T cells during a 4-day co-culture. Depicted are cytokine levels for 7 donors and *p*-values for linear regression

Matured dendritic cells were harvested and washed before being co-cultured for 4 days with allogeneic T cells in mixed leukocyte reactions. Thereafter, supernatants were assessed for IFNγ production. Capacity of DC to stimulate IFNγ production in allogeneic T cells (Fig. 2c, d) correlated significantly with their ability to produce IL-12p70 (Fig. 3). Finally, DC alone also produced IFNγ but only a fraction of what was produced by T cell co-cultures (Suppl Fig. 1a, b). Thus, from these initial experiments we decided to go on to compare the maturation cocktail containing the GMP-grade poly I:C Hiltonol© in combination with IFNγ and R848, with TNFα alone that has been used by us and others in earlier cancer DC vaccine trials [39–41]. In addition we decided to test both variants with or without the addition of LPS.

### DC matured in presence of IFNγ, R848, and poly I:C express increased levels of co-stimulatory molecules, activation markers, and CCR7

**Fig. 4:**
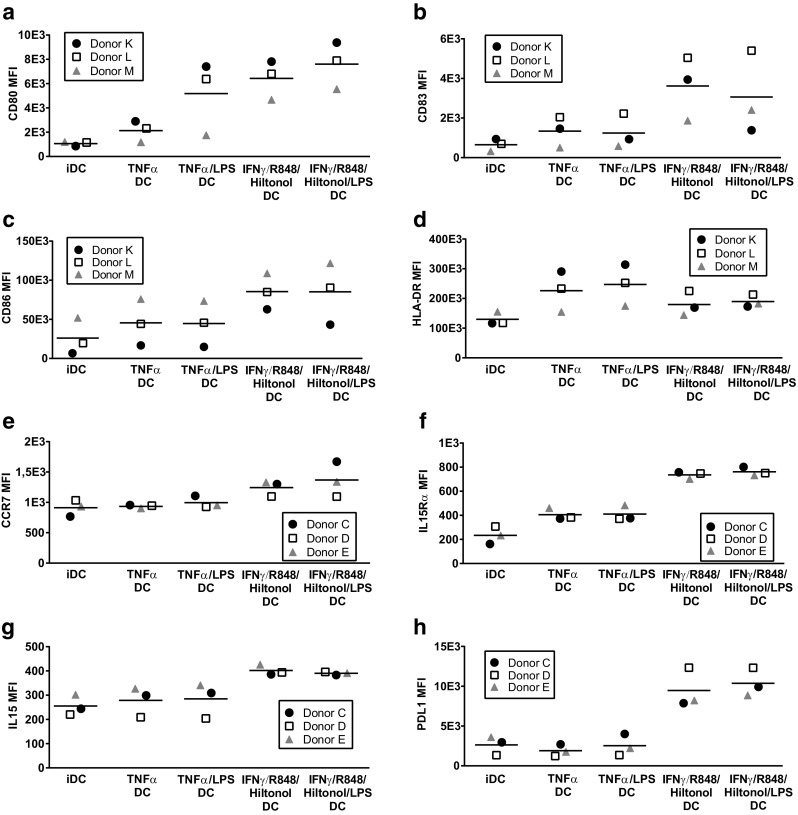
Expression of maturation markers by monocyte-derived dendritic cells matured with different stimulatory cocktails. Monocytes from three donors were cultured for 48 h with GM-CSF/IL-4 followed by harvest, wash, and thereafter 18 h with GM-CSF/IL-4 together with different combinations of TNFα, IFNγ, R848, the GMP-grade poly I:C Hiltonol^®^, and LPS. Thereafter the surface expression of CD80 (**a**), CD83 (**b**), CD86 (**c**), HLA-DR (**d**), CCR7 (**e**), IL-15Rα (**f**), surface-bound IL-15 (**g**), PDL1 (**h**), was assessed by flow cytometry. Depicted values are mean fluorescent intensities

Differently matured DC were analyzed for the expression of surface markers (Fig. 4). All maturation protocols induced upregulation of CD80 (Fig. 4a), CD83 (Fig. 4b), CD86 (Fig. 4c), HLA-DR (Fig. 4d), IL-15Rα (Fig. 4f), and membrane-bound IL-15 (Fig. 4g) compared to iDC. However, upregulation of CD80, CD83, CD86, and IL-15Rα expression as well as increase in membrane-bound IL-15 in DC was much stronger in DC when matured with IFNγ, R848, and Hiltonol© than with TNFα. Furthermore, upregulation of CCR7 (Fig. 4e) was only achieved with cocktails containing IFNγ, R848, and Hiltonol©. On the other hand, HLA-DR expression was increased by TNFα DC but only marginally by combinations containing IFNγ, R848, and Hiltonol© (Fig. 4d). In addition, presence of the co-inhibitory molecule PD-L1 was only induced by IFNγ, R848, and Hiltonol©-containing cocktails (Fig. 4h). The effect of different maturation alternatives on the expression of CD14, DC-SIGN, mannose receptor, and CD40 was variable between individuals (results not shown). Furthermore, presence of LPS in cocktails had limited effect on the expression of DC surface markers, and the effect varied between donors.

### Specific activation of T cells by peptide-loaded DC following maturation in the presence of IFNγ, R848, poly I:C, and LPS

**Fig. 5:**
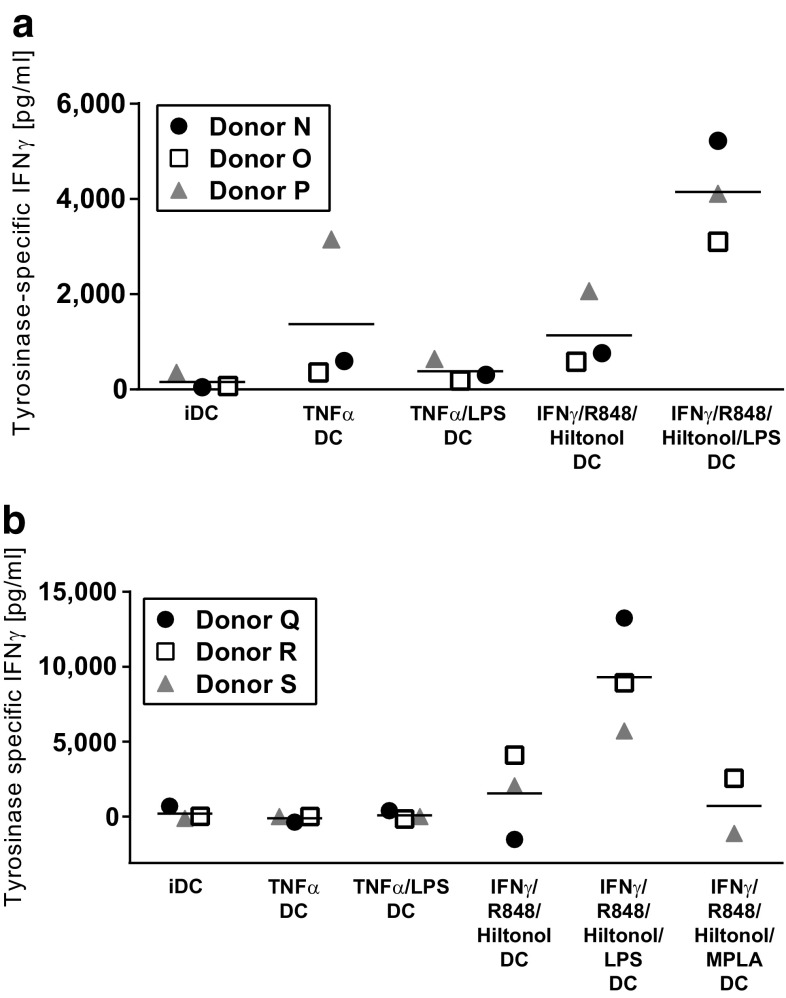
Activation of tyrosinase-specific T cells by tyrosinase-loaded monocyte-derived dendritic cells matured in the presence of different stimulatory cocktails. Monocytes from six donors were analyzed for the production of IL-12p70 after an initial 48 h culture with GM-CSF/IL-4 followed by harvest, wash, and thereafter 18 h with GM-CSF/IL-4 together with the combinations of IFNα, TNFγ, R848, GMP-grade poly I:C Hiltonol^®^, different forms of LPS, and/or monophosphoryl Lipid A (MPLA). The mature monocyte-derived dendritic cells from the first three donors were then harvested, washed, and either kept unloaded or pulsed with tyrosinase or HCV peptide and co-cultured with allogeneic tyrosinase-specific TCR-transduced T cells. Specific activation was calculated by subtracting the IFNγ response against the control HCV peptide-loaded DC from the IFNγ response against the tyrosinase peptide-loaded DC (**a**). For the last three donors, tumor cell lysate from either tyrosinase-negative A375 cells or tyrosinase-transduced A375 was added during the maturation from immature to mature dendritic cells. Thereafter the dendritic cells were harvested, washed, and co-cultured with the tyrosinase-specific T cells. Specific activation was calculated by subtracting the IFNγ response against the control A375 lysate-loaded DC from the IFNγ response against the tyrosinase-expressing A375 lysate-loaded DC (**b**)

Dendritic cell stimulation of T cells transduced with a TCR specific for a tyrosinase-derived peptide presented in HLA-A2 was used as a model for specific T cell activation. Monocytes were purified from HLA-A2-positive blood donors and matured as described. Production of IL-12p70 corresponded well with previous experiments and DC matured in the cocktail combining IFNγ, R848, Hiltonol©, and LPS was clearly the most efficient IL-12p70 producers (Suppl Fig. 2a). After maturation, generated DC were either left unloaded, loaded with tyrosinase peptide, or a control HCV peptide. Next, DC were co-cultured with tyrosinase TCR-transduced T cells for 4 days before IFNγ production was analyzed. Since these T cells in addition to the transduced TCR have an endogenous TCR, there is alloreactivity against unloaded donor DC. Thus, in keeping with previous data, levels of IFNγ produced in co-cultures of T cells and unloaded DC corresponded well with the IL-12p70 produced by the DC, being highest with DC matured in the presence of IFNγ, R848, Hiltonol©, and LPS (Suppl Fig. 3a). This alloreactivity was in most cases slightly decreased when HCV control peptide was added, probably due to competitive binding to MHC molecules (Suppl Fig. 3c). Finally, compared to alloreactive responses, IFNγ production was increased in response to tyrosinase peptide-loaded DC (Suppl Fig. 3e, DC controls: Suppl Fig. 2c, T cell controls: Suppl Fig. 3 g). To calculate the amount of specific activation of T cells, background IFNγ produced in response to control HCV peptide-loaded DC was subtracted from that produced in response to tyrosinase peptide-loaded DC (Fig. 5a). Results showed that only DC matured with IFNγ, R848, Hiltonol©, and LPS could efficiently activate peptide-specific IFNγ production in tyrosinase-specific T cells.

### DC matured in presence of IFNγ, R848, poly I:C, and LPS are able to process and present whole antigen and activate specific T cells

Next, ability of the matured DC to process and present whole antigen from tumor cells was determined. Cell lysates from tyrosinase-positive or tyrosinase-negative A375 melanoma cells were added to DC during the second maturation step. In these experiments, either LPS from Sigma-Aldrich or VacciGrade™ LPS from InVivogen or VacciGrade™ MPLA-SM (an LPS derivative, purified from Salmonella Minnesota) was used to activate TLR4. Amounts of IL-12p70 produced by DC followed a similar trend as in the previous experiments being highest after IFNγ, R848, Hiltonol©, and LPS maturation (Suppl Fig. 2b). The purity of the LPS did not change the results (results not shown), but it was clear that intact LPS was needed as the LPS derivative MPLA-SM did not increase IL-12p70 production in this setting (Suppl Fig. 2b). A synthetic variant of MPLA was also tested, with no effect on IL-12p70 production (results not shown). Following maturation, antigen-loaded DC were co-cultured with tyrosinase TCR-transduced T cells for 4 days before measuring IFNγ. As observed in previous experiments, strongest alloreactive production of IFNγ in responses to unloaded DC was measured after maturation with IFNγ, R848, Hiltonol©, and LPS (Suppl Fig. 3b). DC loaded with tyrosinase-negative A375 lysate induced very similar levels of IFNγ in T cells as the unloaded DC (Suppl Fig. 3d). However, T cells co-cultured with tyrosinase-positive A375 lysate-loaded DC produced increased levels of IFNγ (Suppl Fig. 3f, DC controls: Suppl Fig. 2d, T cell controls: Suppl Fig. 3 h). Specific T cell activation was measured by subtracting the amount of IFNγ produced by T cells co-cultured with tyrosinase-negative A375 lysate-loaded DC from the amount of IFNγ produced against tyrosinase-positive A375 lysate-loaded DC (Fig. 5b). Our data revealed that only DC matured in the presence of IFNγ, R848, Hiltonol©, and LPS could efficiently induce specific activation of IFNγ production in T cells.

## Discussion

In the current study, we show that DC matured in the presence of a combination of IFNγ and ligands for TLR3 (poly I:C), TLR4 (LPS), and TLR8 (R848) display features crucial for triggering efficient T cell-mediated anti-tumor responses. This includes production of pro-inflammatory and Th1-skewing cytokines/chemokines such as IL-12p70, IL1β, IL-6, IL-8, IP-10, TNFα, and type 1, 2, and 3 IFN and increased expression of co-stimulatory molecules such as CD80, CD83, and CD86, the cytokine receptor IL-15Rα as well as surface-bound IL-15, and the lymph node homing receptor CCR7. Most importantly, these DC had the ability to activate T cells unspecifically through allogenic stimulation or specifically via peptide loading or via engulfment of antigen-containing tumor cell lysates.

DC matured in the presence of IFNγ, TLR3, and TLR8 agonists but without LPS expressed similar levels of all surface markers as when LPS was added, but produced much lower levels of most cytokines and did not activate allogeneic or specific T cells to the same degree. The fact that the most efficient maturation mixture contained LPS could be problematic since remaining LPS in the DC vaccine may induce septic shock. We attempted to exchange LPS by MPLA, a LPS derivative reported as less toxic [23, 42], but could not reproduce the results found with intact LPS. Therefore, we believe that, in this setting, LPS has to be included to produce a vaccine able to activate tumor-specific T cells. However, it is essential that the vaccine is thoroughly washed and proven free of endotoxins by appropriate tests before administration to patients. Previous clinical trials have shown that it is feasible to produce a DC vaccine free from endotoxin, even when LPS is included in the production protocol, and that administration to patients was well tolerated [34, 43].

In addition to the vaccine being LPS free, it is equally important that all reagents used are of a high purity to avoid other contaminants. To our knowledge there is no GMP-grade LPS or R848 available in the market. However, for LPS we could show that the VacciGrade™ LPS reagent from Invivogen was as potent as normal grade LPS, and we also used VacciGrade™ R848. These products are produced under aseptic conditions and have been tested to ensure that they activate only the correct TLR pathway. On the other hand, we used different variants of poly I:C with variable results. We found that non-GMP-grade poly I:C was occasionally contaminated with LPS. It is possible that the results of some of the previous reports on DC matured in the presence of non-GMP-grade poly I:C were affected by contaminating LPS. A comparison between the IFNγ, R848, Hiltonol©, and LPS cocktail and the previously used “gold standard” (TNFα, IL1β, IL-6, PGE2) and “alpha type-1 DC” (TNFα, IL-1β, IFNα, IFNγ, Hiltonol©) maturation cocktails showed that both latter cocktails induced only low amounts of IL12p70. This is in contrast to previous reports showing enhanced production of IL12p70 by “alpha type-1 DC” compared to “gold standard DC.” The reason may be that we used the GMP-grade Hiltonol© in the alpha-type-1 DC cocktail instead of the non-GMP-grade poly I:C used in previous publications. Furthermore, poly I:C contains different lengths of dsRNA, varying between manufacturer and batches, and this might affect the uptake and/or downstream signaling. There are several cytosolic dsRNA receptors, e.g., mda-5 and RIG-I, that in addition to the endosomal TLR-3 bind to poly I:C and activate downstream signaling. However, the length of the Poly I:C determines which of these receptors are triggered [44–46]. Since we aimed to have a stable DC maturation protocol we decided to work with the long-strand, GMP-grade poly I:C Hiltonol© which is also less prone to degradation due to addition of a poly-lysine chain.

We have recently treated late-stage melanoma patients with a combination of ACT of TIL and vaccination with tumor lysate-loaded DC [47]. This was a small phase I trial that cannot be used to draw conclusion about clinical efficacy, but one patient responded during the DC vaccination with tumor regression which is still ongoing more than 5 years later and two patients experienced stable disease for 20 and 10 months. There, we used a Mo-DC vaccine differentiated to iDC by IL-4 and GM-CSF and to mDC by TNFα alone simultaneous to loading with autologous tumor lysate. Here, we compare that maturation protocol with the protocol containing IFNγ, R848, and poly I:C. Both protocols were tested with or without LPS. TNFα-matured DC had poor capacity to activate T cells, even when LPS was included. This despite that they most efficiently upregulated HLA-DR and had the lowest upregulation of inhibitory PD-L1. DC matured with IFNγ, R848, and poly I:C cocktails (with or without LPS), on the other hand, expressed medium levels of HLA-DR and high levels of PD-L1, but had strong T cell-activating capacity, especially with LPS added. Furthermore, there was either no effect or a lowered response by including TNFα in the IFNγ, R848, and poly I:C cocktails. Thus, there is no benefit of adding TNFα during DC maturation.

Numerous DC maturation protocols have been previously published by other groups. In comparison with these, the IFNγ, R848, poly I:C, and LPS cocktail-matured DC rank among the best when it comes to the amount of IL-12p70 produced and the ability to activate IFNγ production in T cells. Previous studies have shown a synergistic effect on DC IL-12p70 production by including IFNγ [9, 19, 24] or by combining several TLR ligands [24, 26, 27]. One study showed that melanoma patient DC matured with CD40L and IFNγ were often deficient in IL12p70 production but that this could be counteracted by addition of the two TLR ligands R848 and poly I:C [28] and that vaccination of melanoma patients with such CD40L/IFNγ/R848/poly I:C-matured DC loaded with neoepitope and shared peptides resulted in in vivo expansion of specific T cells [48]. Furthermore, another study reported improved anti-tumor activity and increased resistance to immunosuppression by tumor-reactive T cells expanded in vitro in the presence of anti-CD3 and allogeneic, IFNγ, R848, and poly I:C-matured DC compared to in the presence of anti-CD3 and allogeneic, irradiated bulk PBMC [49]. However, to our knowledge this is the first time a DC maturation cocktail with IFNγ and three TLR ligands are used. In line with the previous publications, our data clearly show that the presence of IFNγ during maturation potentiates the IL-12p70 production and thereby the T cell-activating ability of the DC. Furthermore, addition of three TLR ligands in the maturation cocktails is more efficient than two TLR ligands for generating DC with high IL-12p70, but still modest IL-10, production, and strong T cell-activating capacity. In line with this, DC loaded with whole tumor cell lysates and matured in the presence of IFNγ and three, but not two, TLR ligands were shown to convincingly activate tumor antigen-specific T cell responses. Whether the increase in response is due to the precise TLR ligands used or just an additive effect of using several ligands has not been elucidated. However, it is clear that there is a great variation between individuals in the ability to respond to a certain TLR ligand. Using several TLR ligands should therefore increase the likelihood to generate efficient Mo-DC in most individuals. For example, in our experiments the addition of poly I:C has the smallest impact on IL12p70 production in most donors, but in donors where the LPS response is weak poly I:C strongly increased the amount of IL12p70 produced by DC and the IFNγ produced by T cells in corresponding co-cultures (see donor F in Fig. 2). Thus, in future cancer DC vaccine clinical trials the maturation of the DC should be performed in the presence of IFNγ, R848, poly I:C, and LPS.

## Electronic supplementary material

Below is the link to the electronic supplementary material.

Supplementary material 1 (PDF 286 kb)

## Abbreviations

DAMP: Damage-associated molecular patterns
dsRNA: Double-stranded RNA
HSA: Human serum albumin
iDC: Immature DC
mDC: Mature DC
Mo-DC: Monocyte-derived DC
MPLA-SM: Monophosphoryl lipid A from Salmonella Minnesota
PAMP: Pathogen-associated molecular patterns
PRR: Pattern recognition receptors
PAP: Prostatic acid phosphatase
PGE2: Prostaglandin E2
ssRNA: Single-stranded RNA
