# Supplementary material for: Enhanced stimulation of human tumor-specific T cells by dendritic cells matured in the presence of interferon-γ and multiple toll-like receptor agonists
Source: Cancer Immunol Immunother. 2017 Jun 10;66(10):1333–44. doi: 10.1007/s00262-017-2029-4 (PMC5626805; doi:10.1007/s00262-017-2029-4)
Supplement: Supplementary file 1 — Supplementary material 1 (PDF 286 kb) [file 262_2017_2029_MOESM1_ESM.pdf]

Supplementary figure 1.

a.

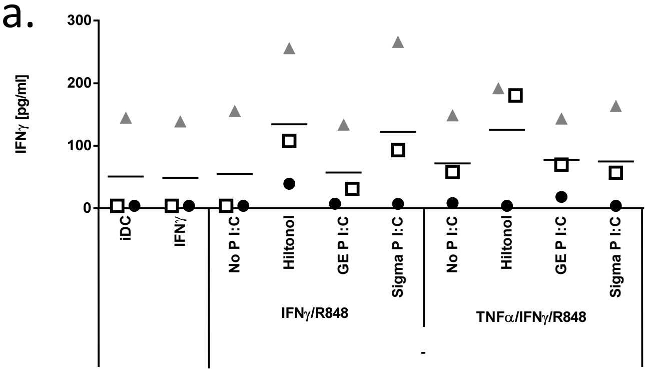

b.

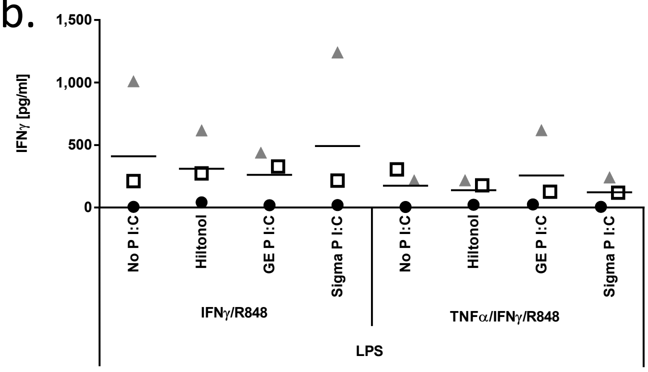

Supplementary figure 1. IFNγ-production by DC matured by different maturation cocktails. Monocytes from three donors were analyzed for production of IFN γafter an initial 48 hours culture with GM-CSF/IL-4 followed by 18 hours with GM-CSF/IL-4/IFNγ/R848 together with combinations of TNF αand GMP-grade poly I:C Hiltonol® or non-GMP grade poly I:C from either GE Healthcare or Sigma-Aldrich without **(a)** or with addition of LPS **(b)**, and finally 4 days culture in 20 IU/ml IL-2 and 2% human AB serum. These DC were analyzed as a control to DC/T cell co-cultures.

Supplementary figure 2

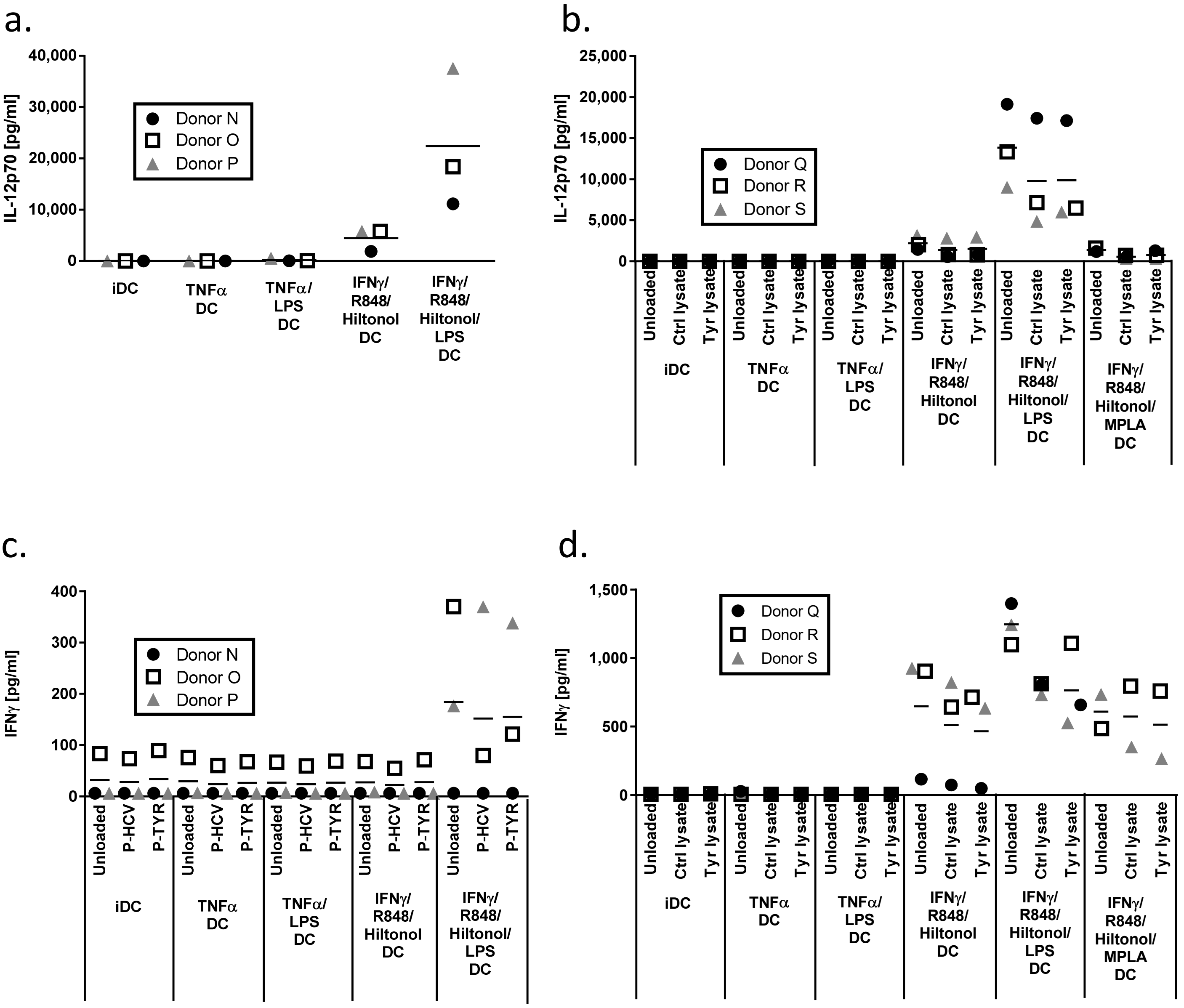

Supplementary figure 2. IL12p70 and IFNγ-production by DC matured by different maturation cocktails. Monocytes from six donors were analyzed for production of IL12p70 (**a** – 3 donors, **b** – 3 donors) after an initial 48 hours culture with GM-CSF/IL-4 followed by harvest, wash and thereafter 18 hours with GM-CSF/IL-4, different combinations of TNFα, IFNγ, R848, Hiltonol, LPS, monophosphoryl Lipid A (MPLA) and tumor cell-lysates from cells with or without tyrosinase expression (only **b**). Thereafter the mature DC were harvested, pulsed with HCV or tyrosinase peptides (only **c**) and further cultured for 4 days culture in 20IU/ml IL-2 and 2% human AB serum before IFNγ levels were measured (**c** – 3 donors,**d** – 3 donors). These DC were analyzed as a control to DC/T cell co-cultures .

Supplementary figure 3

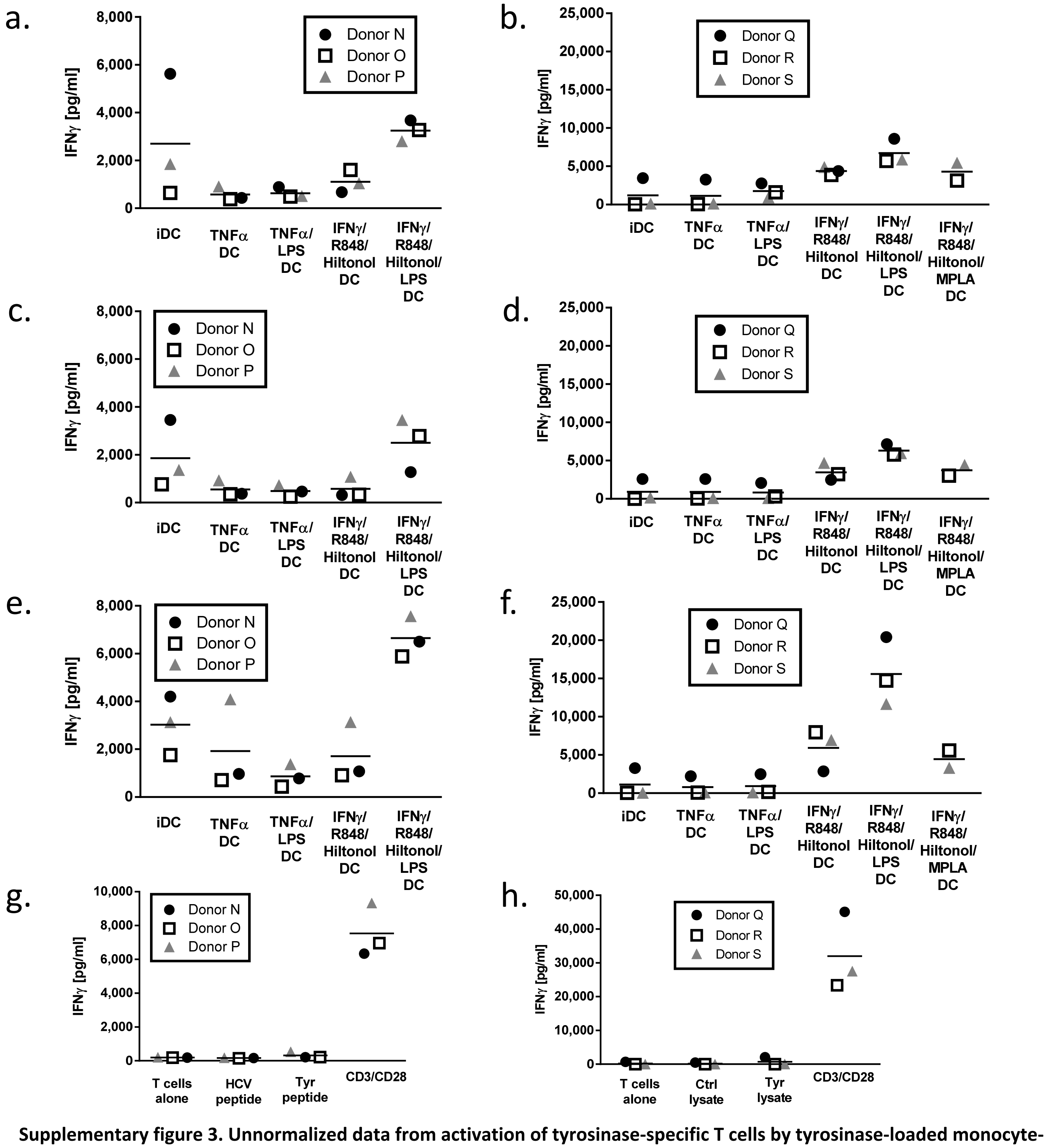

Supplementary figure 3. Unnormalized data from activation of tyrosinase-specific T cells by tyrosinase-loaded monocyte-derived dendritic cells matured in presence of different stimulatory cocktails. Monocytes from six donors were cultured 48 hours culture with GM-CSF/IL-4 followed by harvest, wash and thereafter 18 hours with GM-CSF/IL-4 and combinations of IFNγ, TNFα, R848, GMP-grade poly I:C Hiltonol®, LPS and/or monophosphoryl Lipid A (MPLA) before being harvested and washed once again. The monocyte-derived dendritic cells from the first three donors were kept unloaded **(a)** or were pulsed with HCV **(c)** or tyrosinase **(e)** peptide and co-cultured with tyrosinase-specific allogeneic T cells before analyzing IFN γlevels. The monocyte-derived DC from the last three donors were kept unloaded **(b)** or were loaded with tumor cell lysate from either tyrosinase-negative A375 **(d)** cells or tyrosinase-transduced A375 **(f)** during the maturation from immature to mature dendritic cells. The dendritic cells were co-cultured with allogeneic tyrosinase-specific T cells and IFN γproduction was analyzed. The T cells were also cultured alone, with directly added HCV/tyrosinase peptide or lysates from control and tyrosinase-expressing tumor cells or with CD3/CD28 beads **(g, h)**.
